# Manipulating Self-Avatar Body Dimensions in Virtual Worlds to Complement an Internet-Delivered Intervention to Increase Physical Activity in Overweight Women

**DOI:** 10.3390/ijerph17114045

**Published:** 2020-06-05

**Authors:** Jessica Navarro, Ausiàs Cebolla, Roberto Llorens, Adrián Borrego, Rosa M. Baños

**Affiliations:** 1 Department of Personality, Evaluation and Psychological Treatment, University of Valencia, 46010 Valencia, Spain; ausias.cebolla.marti@gmail.com (A.C.); Rosa.Banos@uv.es (R.M.B.); 2 CIBER Physiopathology of Obesity and Nutrition (CIBEROBN), Carlos III Institute, 28029 Madrid, Spain; 3 Neurorehabilitation and Brain Research Group, Instituto de investigación e Innovación en Bioingenieria, Universitat Politécnica de Valencia, 46022 Valencia, Spain; rllorens@i3b.upv.es (R.L.); aborrego@lableni.com (A.B.); 4 NEURORHB, Servicio de Neurorrehabilitación de Hospitales Vithas, 46007 Valencia, Spain; 5 Polibienestar Institute, 46022 Valencia, Spain

**Keywords:** physical activity, overweight, virtual reality, virtual environments, avatars, intervention

## Abstract

Virtual reality has been found to be a useful tool for positively influencing relevant psychological variables in order to increase physical activity (PA), especially in the overweight population. This study investigates the use of avatars and their physical variations to extend the effectiveness of existing interventions to promote PA. The main objective is to analyze the influence of the avatars’ body dimensions on the efficacy of an Internet intervention to increase PA levels and improve other relevant variables (motivation toward PA, enjoyment, anxiety, self-efficacy, and PA goals). A total of 42 overweight women received a brief online intervention, and they were randomly assigned to one of three conditions: the “Ideal avatar” (IAC: participants are represented by avatars with ideal body dimensions); the “Real avatar” (RAC: participants are represented by avatars with participants’ current body dimensions); and the “Non avatar” (NAC: participants are not represented by avatars). Results showed that the online intervention was effective in increasing PA practice and self-efficacy expectations. However, manipulating the body dimensions of avatars did not improve this intervention, although ideal avatars helped to reduce the anxiety experienced during PA in this population.

## 1. Introduction

Physical inactivity and sedentariness are considered serious health problems with great economic, social, and individual impact [1]. National and worldwide associations and institutions have proposed a series of recommendations for the minimum amount of physical activity (PA) required for health [2]. However, the majority of overweight people do not meet these minimums [3]. 

Empirical evidence has shown relationships between low PA levels and several psychological variables, such as low self-efficacy expectations, low motivation, low enjoyment, negative body representations, or anxiety during PA, among others [4,5,6,7,8,9,10,11,12,13,14]. In addition, different interventions have been designed to change this tendency, including Internet-delivered interventions [15]. These interventions have been found to increase PA motivation in normal and overweight populations [15], although their long-term effectiveness has not been established [15,16].

Other technologies, such as virtual reality (VR), have also been proposed as helpful tools for learning healthy behaviors, such as PA habits [17,18], and there is evidence that virtual experiences can promote PA practice [18,19,20].

The use of VR has several advantages, such as the ability to manipulate body representations [21,22,23] and increase self-efficacy expectations, motivation, or adherence. For instance, some studies have shown that using avatars that physically resemble the user can increase expectations of self-efficacy toward PA and motivate adherence to the practice [18,24] in normal-weight individuals. These results can be explained by Bandura’s social cognitive theory [25], which assumes that individuals vicariously learn new behaviors by observing these behaviors in others [25]. In VR scenarios, avatars can have a strong physical resemblance to individual users [26], and individuals are more likely to learn a behavior if they identify with the model [25].

Regarding the overweight and body dissatisfied population, research shows that avatar resemblance increases awareness of a negative body image and anxiety during PA practice. Song and colleagues [23] found that when participants with body image dissatisfaction embodied avatars representing their ideal body, they showed greater enjoyment and decreased anxiety levels during PA practice. Therefore, using avatars with different body dimensions could help individuals to overcome body representation difficulties during PA practice [21,22] and, consequently, encourage them to exercise [23]. 

Several studies have pointed out that manipulation of the body dimensions of virtual avatars can influence PA practice [24,27,28,29]. Specifically, participants embodying normal-weight avatars showed more PA on a virtual task, compared to overweight avatars [27,28,29]. These results can be explained by the “Proteus effect” [30], which assumes that individuals change their behavior in accordance with the characteristics and appearance of their avatars, in order to conform to the expectations and stereotypes of these avatars. Some research has found support for this effect in different VR experiences [17,31]. 

To date, studies on the influence of avatars on PA practice have consisted of sessions where the avatar’s physical characteristics were manipulated and the impact on the execution of a PA task was analyzed at that moment [24,27,28,29] or in the subsequent practice of PA within a short period of time [18]. However, no studies have tested whether the use of avatars and their physical variations can enhance the effectiveness of existing interventions to increase the level of PA. 

The aim of the present study is to analyze the influence of avatars’ body dimensions on the efficacy of an Internet-delivered intervention specifically designed to increase PA levels in overweight and obese sedentary women. In order to test this objective, participants receive a brief online intervention [32] enriched with a virtual task using avatars. Three conditions are compared, according to the virtual task participants have to perform: (a) the “Ideal avatar condition” (IAC: participants are represented by avatars with body dimensions they rated as “ideal”); (b) the “Real avatar condition” (RAC: participants are represented by avatars with their own current body dimensions); and (c) the “Non avatar condition” (NAC: participants are not represented by avatars while performing PA). The influence of these experimental conditions on several relevant psychological variables (motivation, enjoyment, anxiety, self-efficacy, and PA goals) are analyzed.

We hypothesize that all participants will improve their PA levels after the intervention. In addition, we hypothesize that participants represented by avatars (IAC and RAC conditions) will show a significantly higher PA level and achievement of PA goals than NAC participants. We also hypothesize that IAC and RAC participants will increase their scores on motivation, enjoyment, and self-efficacy, and this increase will be higher than in NAC participants. In addition, we expect that IAC participants will choose more ambitious PA goals and show lower anxiety while performing PA, compared to RAC participants. Finally, we hypothesize that similarity to the avatar and self-efficacy expectations will mediate between the conditions and the increase in PA levels after the intervention. 

## 2. Materials and Methods

### 2.1. Participants

**Figure 1:**
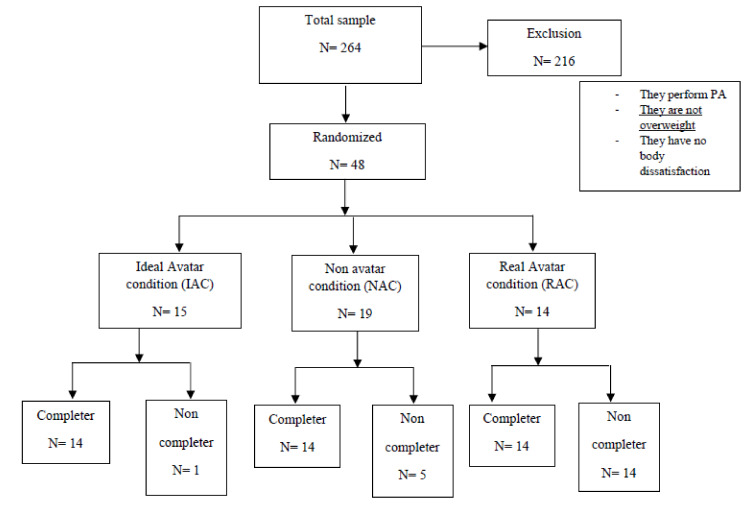
Flow chart of participants’ recruitment.

The final sample was composed of 42 overweight and obese women (BMI, *M* = 28.7; SD = 3.1) who were sedentary and had high body dissatisfaction (see participants’ recruitment flow in Figure 1). Their ages ranged from 19 to 61 years (*M* = 31.9; *SD* = 11.7). Participants were recruited in nutrition clinics and gyms. Because participants had to show low activity levels, the gyms only contacted women who had dropped out. Flyers and in-person presentations were used to publicize the study. The eligibility criteria were: being a woman from 18–64 years old; being overweight (BMI > 25); having high body dissatisfaction (Body Schema Questionnaire—BSQ— > 80); being physically inactive; and not having any physical condition that could keep them from practicing PA. Of the total 216 participants excluded from the study, 70% of the them were excluded because they were not overweight, and 30% of the remaining participants were excluded for reasons related to PA practice (e.g., they were physically active) or body dissatisfaction (e.g., they showed no body dissatisfaction). Participants were informed about the study, and they signed informed consent documents. This study was approved by the Ethical Committee of the University of Valencia (Spain). 

### 2.2. Procedure

After the participants contacted us, they were informed about the contents of the study by telephone, and they signed the informed consent by email. Participants who met the criteria were randomly assigned to one of the three conditions (IAC: 14; RAC: 14; NAC; 14), using the Random Allocation Software 2.0 (This software has been developed by M. Saghaei, MD., Department of Anesthesia, Isfahan University of Medical Sciences, Isfahan, Iran). First, they were sent an email with the link to fill out the questionnaires online. Then, they received a link with the online intervention they had to follow for a week to increase PA. The specific time spent on this online intervention was registered for each participant. After seven days, participants were individually invited to the laboratory, where the virtual PA task was applied for about 10 min. The virtual task varied in the three conditions.

(a) IAC participants were asked to create an avatar with their ideal body dimensions and their own face. They were shown a default avatar and were able to change its body dimensions. Then, they performed a running task for 4 min in a VR scenario where they were represented by this avatar. The VR task performance was video-recorded, and participants received this video on their mobile phones and were asked to watch it every day of the week.
(b) RAC participants received the same instructions, but they were asked to change the avatar (with their face) to fit their real body dimensions.
(c) NAC participants were asked to perform the PA task in the VR scenario for 4 min, but participants were not represented by an avatar. They ran in front of a fixed image corresponding to the VRE. They did not receive any video-recordings.

Finally, all participants were asked to choose a weekly PA goal (walking or running three times a week). A week later, they received an email with the link to answer the questionnaires online. Finally, all participants came back to the lab to report on the achievement of the PA goals and receive their reward for completing the study (an invitation to a gymnasium where they could participate in sports activities and study their physical condition). All participants were met by blinded study staff. 

### 2.3. Materials

#### 2.3.1. VR Program

The VR scenario consisted of a 3D graphical environment representing a park where an avatar runs. Avatars’ characteristics varied depending on the experimental condition (IAC and RAC). In the IAC and RAC conditions, the participant’s face was tracked by the Kinect. All participants ran in place in a room, and their movements were captured by a Kinect and projected on a 150 × 150 cm screen. During the PA task, participants could see the time and distance they had run on the screen. 

#### 2.3.2. Online Intervention 

This brief one-session intervention is based on the trans-theoretical model components of behavior change [33], and it has shown its effectiveness in previous studies [32]. It consists of two parts: the first one, “Motivation for Change”, provides information on PA, recommendations, consequences of physical inactivity, and possible barriers; the second part, “Move it”, focuses on helping participants to find their own motivation and set their specific PA goals for the future. For a more detailed description, see [32]. The entire intervention lasted about 45 min, and the specific time spent on the intervention screen was recorded for each participant.

### 2.4. Measures

Anthropometric and sociodemographic data. An ad-hoc questionnaire was created to collect information about sociodemographic data, height, and weight.

Body Shape Questionnaire (BSQ [34]). It consists of 34 items, rated on a scale from 1 to 6 (1 = “never” to 6 = “always”), that evaluate the dissatisfaction produced by one’s body, the fear of gaining weight, self-devaluation due to physical appearance, the desire to lose weight, and avoidance of situations where one’s physical appearance could attract the attention of others. The measure is the composite sum of the items, and higher scores reflect greater body dissatisfaction in the past four weeks. There are four categories of concern: “no concern” (<81), “mild concern” (81–110), “moderate concern” (111–140), and “extreme concern” (>140) [34]. The cut-off point for inclusion in this study was 81. The Spanish version used in this study showed adequate internal consistency [35].

International physical activity questionnaire (IPAQ [36]): Through 31 items, this questionnaire collects data on PA performed in the past 7 days. It identifies the frequency and duration of moderate and vigorous leisure, transportation and occupational PA, walking PA, and inactivity during the past week. The IPAQ has reported test-retest reliability correlations of 0.81 and validity correlations with accelerometers of 0.33 [37]. 

Weekly PA goal registration: This ad hoc record collects data on the weekly achievement of the specific PA goal in all the conditions, as well as the video display for the IAC and RAC conditions. The two possible goals were walking or running three times a week. All participants were free to choose one of these two goals. 

Behavioral Regulation in Exercise Questionnaire (BREQ-2 [38]). This questionnaire is based on the theory of self-determination, which provides insight into the reasons people adopt and maintain healthy behaviors [39,40]. It consists of 19 items, rated on a scale from 0 to 5 (0 = “Not at all true for me” to 5 = “absolutely true for me”), that measure stages on the continuum of self-determination in PA behavior. This questionnaire assesses external regulation, introjected regulation, identified regulation, and intrinsic regulation, and it adds demotivation. The BREQ-2 has shown acceptable internal consistency [41]. 

Self-efficacy to regulate exercise (ESE [42]). It consists of 18 items, rated on a scale from 0 to 100 (0 = “not at all sure” to 100 = “Very sure”), that evaluate how sure the person is about regularly performing an exercise routine (three or more times per week). The measure is the composite mean of the items, and higher scores reflect greater PA self-efficacy. This scale has been shown to be a useful measure of exercise self-efficacy expectations in several populations [43,44,45]. 

Enjoyment (PACES [46]): This questionnaire consists of 16 items, rated on a scale from 1 to 5 (1 = “Strongly disagree” to 5 = “Strongly agree”), that evaluate the degree of enjoyment of PA. The measure is the composite mean of the items, and higher scores reflect more enjoyment of PA. PACES has been a useful instrument to measure enjoyment in different fields of PA [47]. 

The physical activity and sport anxiety scale (PASAS [48]). This is a 16-item self-report that assesses social fear and avoidance of sports and PA on a scale from 1 (“not at all characteristic of me”) to 5 (“extremely characteristic of me”). This measure has demonstrated good internal consistency, test-retest reliability, and convergent and divergent validity [48]. 

Avatar identification modified questionnaire [49]. It consists of 17 items, rated on a scale from 1 (“Strongly disagree”) to 5 (“Strongly agree”), that assess the degree of embodied presence, perceived similarity, and the participant’s desire to identify with the avatar. This self-report has been shown to be a reliable measure of identification in online games [49].

### 2.5. Data Analyses 

Statistical analyses were conducted using the SPSS for Windows (version 24) (This software has been developed by Norman H. Nie, Dale H. Bent, and C. Hadlai Hull., University of Stanford, United states). First, to assess the influence of the avatars’ body dimensions on PA, repeated-measures ANOVA were performed on each variable (motivation, enjoyment, anxiety, self-efficacy, and PA levels), with condition (3: IAC, RAC, and NAC) as between factor and time (2: pre versus post intervention) as within factor. In addition, univariate ANOVAs were carried out to analyze the differences between conditions in the time spent on the intervention, video display during the week, and the achievement of PA goals. When a significant interaction was found, post-hoc analyses using Bonferroni adjustment were conducted to determine which group comparisons were significant. 

Second, to check differences between conditions in the PA goal chosen, a chi-square test was performed, using Monte Carlo with 10,000 samples as a 99% level of confidence. When the absolute value of the adjusted standardized residual was greater than 1.96, there were significant differences between conditions. Subsequently, effect sizes (Cohen’s *d)* and confidence intervals were calculated for within-group changes, given that effect sizes are the best indicator of the magnitude of the observed changes, which is essential information that cannot be obtained by focusing exclusively on *p*-values [50].

Finally, using Model 6 from PROCESS 3.3, we performed two serial multiple mediation analyses to test whether the effects of condition on the change in PA were mediated by self-efficacy and perceived similarity to the avatar. The procedure described by Hayes [51] was performed using the PROCESS macro for SPSS. Significance tests (*p* < 0.05) or a confidence interval (not including zero) for the interaction answered this question. 

## 3. Results

### 3.1. Adherence to Tasks

Time spent on the online intervention. Descriptive statistics can be found in Table 1. For the time spent on the intervention, the results showed a wide range from 1 to 372 min (*M* = 46.71; *SD* = 68.49). Results did not show differences between conditions *F* (2, 40) = 0.25, *p* = 0.779, η = 0.01. 

Watching the avatar video during the week. Descriptive statistics can be found in Table 1. Most of the participants watched the video daily (*M* = 5.96; *SD* = 1.55). There were no differences across conditions *F* (1, 27) = 0.36, *p* = 0.552, η = 0.01. 

### 3.2. Efficacy Results: Differences between Conditions 

Descriptive statistics and within-group effect sizes (measured by Cohen’s *d*) can be found in Table 2. 

PA levels (IPAQ [37]): Regarding the ANOVA results, there was a main effect of time on PA levels *F* (1, 39) = 15.82, *p =* 0.000, η = 0.29. All participants showed higher PA levels after the intervention. However, the interaction between time and condition was not significant *F* (1, 39) = 0.05, *p* = 0.949, η = 0.00. 

PA goals (walking or running three times a week). No significant effects were found on the achievement of the PA goal *F* (2, 41) = 0.36, *p* = 0.702, η = 0.02. Regarding the specific PA goal chosen, despite the trends found, chi-square analyses showed no differences between conditions χ2 (2, *N* = 42) = 3.20, *p* = 0.202, Cramer’s V = 0.28. The percentages of the specific PA goals chosen in each condition are shown in Table 3. The specific PA goal of walking was chosen by 73.8% of the participants.

Motivation toward PA (BREQ-2 [38]). No effect of time was found on any subscale (intrinsic regulation *F* (1, 39) = 0.12, *p* = 0.913, η = 0.00, identified regulation *F* (1, 39) = 1.65, *p* = 0.207, η = 0.04, introjected regulation *F* (1, 39) = 1.10, *p* = 0.300, η = 0.03, external regulation *F* (1, 39) = 1.93, *p* = 0.173, η = 0.05, and demotivation *F* (1, 39) = 3.57, *p* = 0.066, η = 0.08). No interactions between time and condition were significant for any subscale (intrinsic regulation *F* (2, 39) = 2.76, *p* = 0.076, η = 0.12, identified regulation *F* (2, 39) = 1.09, *p* = 0.347, η = 0.05, introjected regulation *F* (2, 39) = 0.31, *p* = 0.736, η = 0.02, external regulation *F* (2, 39) = 0.21, *p* = 0.811, η = 0.01, and demotivation *F* (2, 39) = 2.07, *p* = 0.139, η = 0.09). 

Enjoyment (PACES [46]). No time effect was found on enjoyment (*F* (1, 39) = 0.63, *p* = 0.432, η = 0.02), and the interaction effect between time and condition was not significant either (*F* (2, 39) = 0.25, *p* = 0.776, η = 0.01).

Anxiety (PASAS [48]). There was a main effect of time on total anxiety during PA practice (*F* (1, 39) = 18.18, *p* = 0.000, η = 0.32). All participants showed lower anxiety levels during PA after the intervention. In addition, the interaction between time and condition was also significant (*F* (2, 39) = 4.57, *p* = 0.016, η = 0.19). Post- hoc comparisons using Bonferroni correction revealed that IAC and NAC participants showed lower anxiety levels during PA after the intervention (*p* = 0.010 and *p* = 0.000), compared to RAC participants. 

Self-efficacy (ESE [42]). There was a main effect of time on self-efficacy toward PA (F (1, 39) = 8.49, *p* = 0.006, η = 0.18). However, despite the trends found, the interaction between time and condition was not significant (F (2, 39) = 0.99, *p* = 0.380, η = 0.05). 

### 3.3. Similarity to the Avatar and Self-Efficacy Expectations as Mediators: Do Similarity to the Avatar and Self-efficacy Influence PA Practice? 

Two serial multiple mediation analyses were carried out to test whether the effects of condition on the change in PA (PA levels and achievement of PA goals) were mediated by similarity to the avatar and self-efficacy expectations.

Regarding the effects on the achievement of the PA goal (Figure 1), the indirect effect of “Condition → change in similarity to the avatar → achievement of PA goal was significant, implying that similarity to the avatar mediated the relationship between the condition and achievement of the PA goal, *b* = −0.40, *SE* = 0.25, 95% CI [−1.15, −0.07]. This result means that participants who perceived the avatar as similar to themselves showed greater achievement of the PA goal. In contrast, the other two indirect effects tested in this serial multiple mediation model were not significant: (a) Indirect effect of “Condition → change in self-efficacy → achievement of PA goal”, *b* = 0.03, *SE* = 0.18, 95% CI [−0.14, 0.62]; (b) Indirect effect of “Condition → change in similarity to the avatar → change in self-efficacy → achievement of PA goal”, *b* = −0.05, *SE* = 0.09), 95% CI [−0.48, 0.01]. 

**Figure 2:**
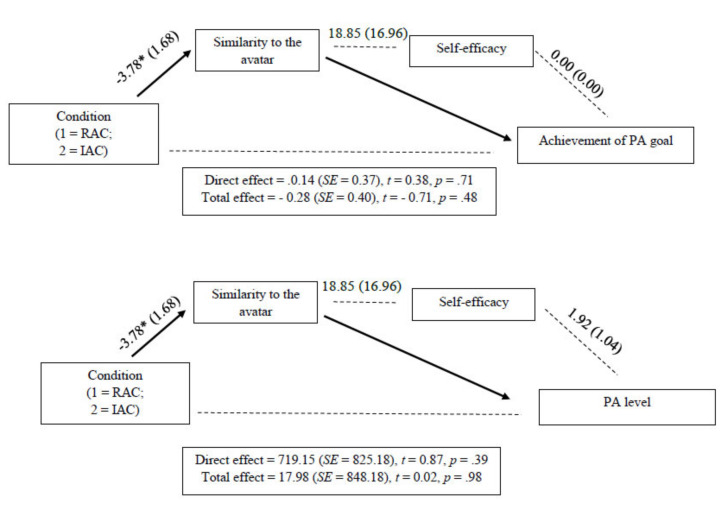
Serial multiple mediation analysis. Note. * *p* < 0.05.

Regarding the effects on PA levels (Figure 2), none of the indirect effects were significant: (a) Indirect effect of “Condition → change in similarity to the avatar → changes in PA levels”, *b* = −646.29, *SE* = 588.49, 95% CI [−2142.42, 103.55]: (b) Indirect effect of “Condition → change in self-efficacy → change in PA levels”, *b* = 82.18, *SE* = 304.67, 95% CI [−692.18, 563.01]: (c) Indirect effect of “Condition → change in similarity to the avatar → change in self-efficacy → change in PA levels, *b* = −137.06, *SE* = 163.95, 95% CI [−512.75, 131.89]. 

## 4. Discussion

This study was conducted to analyze the influence of avatars’ body dimensions on the efficacy of an online intervention to increase PA levels, as well as the influence on other relevant variables (motivation toward PA, enjoyment, anxiety, self-efficacy, and PA goals), in a sample of overweight and body dissatisfied women. A second aim was to explore whether the effects of the condition on the change in PA (PA levels and achievement of PA goals) were mediated by the similarity to the avatar and self-efficacy expectations.

The first hypothesis assumed that the intervention would be effective in increasing PA levels in all participants, regardless of the condition. This hypothesis was confirmed because significant increases were found in PA levels and self-efficacy expectations after the intervention in all participants. Previous studies with this intervention showed its effectiveness in a sample of university students [32]. Specifically, previous results revealed that it had been effective in increasing awareness of the positive consequences of PA practice, influencing the strategies used to modify the PA habit, increasing enjoyment during PA practice, and, consequently, increasing the number of weekly steps. The results of this study confirm the efficacy of this brief online intervention in overweight and obese populations. These data are quite promising because it is a very short, self-applied intervention and can be quite cost effective in increasing PA in different populations. It would be interesting to include a follow-up measure to show the medium- and long-term effects of this intervention. 

The second hypothesis proposed that participants represented by avatars (IAC and RAC) would show higher levels of PA, achievement of goals, motivation, self-efficacy, and enjoyment, compared to NAC participants. In addition, we expected that IAC participants would choose more ambitious goals and show lower anxiety while performing PA because using avatars with different body dimensions has been shown to help individuals to overcome body representation difficulties during PA practice [21,22]. This hypothesis was only confirmed for anxiety scores. As expected, the IAC participants showed lower levels of anxiety compared to RAC participants. However, it is important to highlight that the NAC participants scored the lowest on anxiety. NAC participants were not represented by any avatar or exposed to their body during the virtual PA task, and they performed the PA task in front of an image of a park, which could act as a distracting stimulus from their own body. Our results suggest that the use of avatars in virtual scenarios can elicit anxiety in overweight and body dissatisfied individuals by increasing self-body image awareness [23], and this anxiety induction could be higher with avatars representing their real body dimensions rather than ideal body dimensions. According to the literature, dissatisfaction with body image acts as a barrier to PA practice [9,10] especially in contexts where body image is more prominent, such as group PA or mirror environments [11,13,14,52]. Our results seem to support this line of research. The use of avatars in the context of overweight and dissatisfied women may enhance the anxiety experienced towards PA since the avatar highlights the individual’s body image, especially when the avatar represents the real self.

Regarding the lack of significant differences in PA levels and the goal chosen across conditions, our manipulation failed to bring about a major change in PA levels or the choice of a more ambitious goal in IAC participants. First, a high percentage of participants (61.9%) reported that they had successfully achieved their objective. Because they reported this achievement at a face-to-face meeting, social desirability may have had an effect on this report [53]. Perhaps it would have been preferable to record the achievement online rather than in a face-to-face visit. Regarding the choice of the PA goal, 73.8% of the participants chose to walk, that is, the less ambitious goal. These results are not surprising, as the evidence shows that brisk walking is the preferred PA type for overweight women [54]. Therefore, it would have been more appropriate not to compare two such different objectives (running versus walking) but to measure the intensity of the PA performed, for example, by providing participants with an accelerometer during the PA practice that measures the intensity of walking. It seems that manipulating the avatar while performing the intervention has no effect on promoting a more ambitious goal in the participants.

Despite the lack of differences, it is important to highlight the changes observed in the RAC condition. According to standardized effect sizes (Cohen’s *d*), this group obtained a large effect size (>0.80) for their change in PA levels, and they increased their weekly practice the most. Similarly, the results also showed: a medium effect size (<0.50) for intrinsic motivation in the RAC participants, who increased their intrinsic motivation scores the most; a large effect size (>0.80) for external motivation in the IAC participants, who increased their external motivation scores the most; and a large effect size (>0.80) for self-efficacy expectations in participants in the NAC condition, who increased their self-efficacy expectations scores the most. Given these results, although through this study no significant differences between the groups can be concluded, it would be interesting to increase the statistical power of the study. It is possible that increasing the sample size, greater differences might be found. 

The third hypothesis proposed that similarity to the avatar and self-efficacy expectations would mediate between the condition and PA practice. This hypothesis was partially confirmed in the case of achievement of the PA goal. Participants who had judged their avatar to be more similar to themselves were more likely to reach the PA goal. These results are in line with the literature, showing that virtual self-models can be effective instigators of PA change [18]. In general, research shows that when individuals personalize their avatars they self-report higher behavioral intentions, as measured by the percentage of time they intended to spend on maintaining good health [55,56]. However, intentions and actual behavior associated with such intention did not always correlate and results did not always go in the same direction [57], which could explain the absence of differences in weekly PA levels. The lack of significance of self-efficacy expectations as a mediator between the condition and the achievement of the PA goal could be due to the characteristics of the sample. Other studies have concluded that, although an increase in self-efficacy in normal weight participants has an impact on PA, in obese or overweight people, this effect is not significant [58]. 

Some limitations of the current study should be mentioned. The first is that, due to technical limitations, NAC participants did not use a dynamic VR scenario, but rather a fixed image. It would be desirable for all participants to use a VR scenario, with or without an avatar. Second, assessments were only carried out before and after the intervention, and it would be preferable to have more assessments moments (e.g., different times throughout the PA task), as well as follow-ups. 

As future lines, it would be desirable to include follow-ups to analyze the long term effects of the intervention as well as to include women without body dissatisfaction. 

## 5. Conclusions

In conclusion, the online intervention used in this study was effective in increasing PA practice and self-efficacy expectations in overweight women. Manipulating the body dimensions of avatars did not improve this intervention. Using ideal avatars seems to reduce the anxiety experienced during PA in this population. However, the use of avatars similar to the person him/herself could have a greater impact on PA and variables related to its long-term practice. 

## Figures and Tables

**Table 1:** ANOVA results for baseline measures and intervention adherence.

Measure	Condition	*N*	M (SD) Baseline	*p*
PA levels	NAC	14	2499.81 (2231.45)	0.571
	RAC	14	1902.33 (971.67)	
	IAC	14	2552.98 (1927.08)	
	Total	42	2318.37 (1773.36)	
Intrinsic Regulation	NAC	14	10.57 (3.41)	0.413
	RAC	14	9.64 (1.82)	
	IAC	14	10.93 (2.34)	
	Total	42	10.38 (2.60)	
Identified Regulation	NAC	14	10.93 (3.22)	0.091
	RAC	14	11.71 (3.27)	
	IAC	14	13.29 (1.64)	
	Total	42	11.98 (2.92)	
Introjected Regulation	NAC	14	9.00 (1.62)	0.423
	RAC	14	8.71 (1.07)	
	IAC	14	9.43 (1.55)	
	Total	42	9.05 (1.43)	
External Regulation	NAC	14	10.57 (3.41)	0.634
	RAC	14	10.71 (1.86)	
	IAC	14	11.36 (0.93)	
	Total	42	10.88 (2.28)	
Demotivation	NAC	14	10.07 (4.32)	0.264
	RAC	14	8.36 (3.65)	
	IAC	14	7.93 (2.67)	
	Total	42	8.79 (3.65)	
Enjoyment	NAC	14	60.64 (11.47)	0.323
	RAC	14	63.79 (10.89)	
	IAC	14	66.86 (9.91)	
	Total	42	63.76 (10.82)	
Anxiety	NAC	14	47.93 (13.08)	0.253
	RAC	14	40.93 (16.34)	
	IAC	14	38.71 (15.65)	
	Total	42	42.52 (15.24)	
Self-efficacy	NAC	14	469.28 (185.45)	0.111
	RAC	14	646.43 (317.29)	
	IAC	14	752.14 (483.23)	
	Total	42	622.62 (361.68)	
Body Mass Index	NAC	14	29.39 (3.57)	0.627
	RAC	14	28.42 (2.69)	
	IAC	14	28.35 (3.22)	
	Total	42	28.72 (3.13)	
Motivational Intervention	NAC	14	38.69 (38.37)	0.779
	RAC	14	57.43 (96.23)	
	IAC	14	44.76 (65.00)	
	Total	42	46.71 (68.49)	
Avatar Video	NAC	14	-----	0.552
	RAC	14	5.79 (1.93)	
	IAC	14	6.14 (1.09)	
	Total	28	5.96 (1.55)	
Body Dissatisfaction	NAC	14	117.07 (23.45)	0.817
	RAC	14	116.57 (30.61)	
	IAC	14	110.86 (31.15)	
	Total	42	114.83 (28.06)	

Note. PA levels (for a week) *=* IPAQ; Intrinsic Regulation = BREQ-2 (20); Identified Regulation = BREQ-2 (20); Introjected Regulation = BREQ-2 (15); External Regulation = BREQ-2 (20); Demotivation = BREQ-2 (20); Enjoyment = PACES (80); Anxiety = PASAS (80); Self-efficacy = ESE (1800); Body Mass Index = BMI; Motivational Intervention = minutes dedicated to the online intervention; Avatar Video = days a week of viewing; Levels of Body Dissatisfaction = BSQ (204). The score in brackets is the maximum score in the questionnaire.

**Table 2:** Descriptive statistics and within-group effect sizes for outcomes.

Measure	Condition	N	M (SD) Pre	M (SD) Post	*p*	Within-Group Effect Size, d [95% CI] Pre-post Intervention
Intrinsic Regulation	NAC	14	10.57 (3.41)	10.29 (2.61)	0.076	0.08 [−0.58, 0.73]
	RAC	14	9.64 (1.82)	11.07 (1.64)		**−0.74 [−1.47, −0.01]**
	IAC	14	10.93 (2.34)	9.93 (2.09)		0.40 [−0.28, 1.08]
Identified Regulation	NAC	14	10.93 (3.22)	11.21 (2.08)	0.347	−0.08 [−0.56, 0.39]
	RAC	14	11.71 (3.27)	13.00 (3.03)		−0.37 [−0.87, 0.13]
	IAC	14	13.29 (1.64)	13.21 (2.78)		0.05 [−0.43, 0.52]
Introjected Regulation	NAC	14	9.00 (1.62)	9.43 (0.85)	0.736	−0.25 [−0.85, 0.35]
	RAC	14	8.71 (1.07)	9.00 (1.30)		−0.26 [−0.86, 0.35]
	IAC	14	9.43 (1.55)	9.43 (1.34)		−0.00 [−0.59, 0.59]
External Regulation	NAC	14	10.57 (3.41)	10.07 (2.09)	0.811	0.14 [−0.46, 0.73]
	RAC	14	10.71 (1.86)	10.50 (1.95)		0.11 [−0.49, 0.69]
	IAC	14	11.36 (0.93)	10.57 (1.34)		**0.80 [0.11, 1.48]**
Demotivation	NAC	14	10.07 (4.32)	7.86 (2.32)	0.139	0.48 [−0.08, 1.04]
	RAC	14	8.36 (3.65)	8.29 (2.37)		0.02 [−0.50, 0.54]
	IAC	14	7.93 (2,67)	7.57 (2.28)		0.13 [−0.39, 0.65]
Enjoyment	NAC	14	60.64 (11.47)	62.71 (12.02)	0.776	−0.17 [−0.73, 0.39]
	RAC	14	63.79 (10.89)	63.43 (9.34)		0.03 [−0.52, 0.58]
	IAC	14	66.86 (9.91)	69.14 (7.37)		−0.22 [−0.77, 0.34]
Anxiety	NAC	14	47.93 (13.08)	37.71 (12.09)	0.016	**0.74 [0.29, 1.18]**
	RAC	14	40.93 (16.34)	40.43 (16.95)		0.03 [−0.29, 0.35]
	IAC	14	38.71 (15.65)	32.57 (16.49)		**0.37 [0.01, 0.73]**
Self-efficacy	NAC	14	469.2857 (185.45015)	760.7143 (355.77574)	0.38	**−1.48 [−2.32, −0.64]**
	RAC	14	646.4286 (317.28675)	775.7143 (416.75882)		−0.38 [−0.96, 0.19]
	IAC	14	752.1429 (483.22918)	852.8571 (389.72236)		−0.20 [−0.75, 0.36]
PA levels	NAC	14	2499.807 (2231.4511)	3884.9214 (2671.75408)	0.949	**−0.58 [−1.14, −0.03]**
	RAC	14	1902.329 (971.6688)	3065.2357 (1924.58215)		**−1.13 [−1.81, −0.44]**
	IAC	14	2552.979 (1927.0837)	3733.8714 (2428.44219)		**−0.58 [−1.13, −0.02]**

Note. Intrinsic Regulation = BREQ-2; Identified Regulation = BREQ-2; Introjected Regulation = BREQ-2; External Regulation = BREQ-2; Demotivation = BREQ-2; Enjoyment = PACES; Anxiety = PASAS; Self-efficacy = ESE; PA levels (for a week) = IPAQ. **Bold letter** = effect of sizes of greater magnitude.

**Table 3:** Chi-square test results.

		NAC	RAC	IAC	Total
Walking	Count	12	11	8	31
	Expected count	10.3	10.3	10.3	30.9
	%	38.7	35.5	25.8	100
	ASR	1.2	0.5	−1.7	--
Running	Count	2	3	6	11
	Expected count	3.7	3.7	3.7	11.1
	%	18.2	27.3	54.5	100
	ASR	−1.2	−0.5	1.7	--

Note. Count = number of participants who choose the PA goal; Expected count = number of participants expected to choose the PA goal; % = percentage of participants who choose the PA goal; ASR = Adjusted standardized residuals.
